# Clinical Assessment of Dairy Goats’ Udder Health Using Infrared Thermography

**DOI:** 10.3390/ani15050658

**Published:** 2025-02-24

**Authors:** Vera Korelidou, Zeljana Grbovic, Dejan Pavlovic, Isidora Simovic, Marko Panic, Anastasios Temenos, Athanasios I. Gelasakis

**Affiliations:** 1 Laboratory of Anatomy and Physiology of Farm Animals, Department of Animal Science, School of Animal Biosciences, Agricultural University of Athens (AUA), Iera Odos 75 Str., 11855 Athens, Greece; vkorelidou@aua.gr; 2 BioSense Institute, University of Novi Sad, 21000 Novi Sad, Serbia; zeljanagrbovic@biosense.rs (Z.G.); dejan.pavlovic@biosense.rs (D.P.); isidora.simovic@biosense.rs (I.S.); panic@biosense.rs (M.P.); 3 School of Rural, Surveying and Geoinformatics Engineering, National Technical University of Athens, 9 Heroon Polytechneiou Str., 15773 Athens, Greece; tasostemenos@mail.ntua.gr

**Keywords:** thermal imaging, goat, udder health monitoring, udder thermogram, chronic mastitis, welfare

## Abstract

This study aimed to evaluate the capacity of infrared thermography (IRT) to be used for the in situ clinical assessment of udder health status across lactation in goats. Two epidemiological studies were designed, with 106 purebred Skopelos goats being prospectively monitored over one lactation in the first study and 132 goats of the same breed from a second farm being enrolled in the second, cross-sectional study. Goats were categorized based on the clinical status of each udder half (healthy, fibrotic, both fibrotic and asymmetric, asymmetric, swollen supra-mammary lymph nodes, and abscessed). Skin surface temperature values of teats (TSST) and udder halves (USST), as well as temperature variations between different types of abscesses, based on the stage of their development (superficial developed, superficial fully mature, drained), and the symmetrical sites of the non-affected udder halves, were evaluated. Mixed linear regression analyses indicated that fibrosis, particularly when accompanied by asymmetry, resulted in a decrease of 0.2, 0.3, and 0.3 °C (*p* < 0.05) and of 0.6, 0.9, and 1.4 °C (*p* < 0.001) in the maximum, mean, and minimum USST, respectively. Recording season significantly affected USST and TSST (*p* < 0.001), with both of them increasing between February and August. Additionally, TSST appeared to be an unreliable indicator of udder health status, while abscesses exhibited distinct temperature patterns based on their developmental stage. It is concluded that IRT has the potential to be used on site for the detection of temperature alterations associated with chronic udder health issues, yet future studies on a larger and more diverse population of goats with various udder health conditions is needed to confirm its extensive applicability under real-world conditions.

## Simple Summary

Regular clinical examination of the udder is necessary for identifying udder health-related issues that may affect animal welfare and productivity, yet it can be laborious when applied in large herds and requires restriction and handling of the animals and special expertise of the examiner. Infrared thermography is a quick, non-invasive, proximal optical sensing technology that can detect changes in the skin surface temperature reflecting underlying tissue blood flow and metabolism. The objective of this study was to examine whether infrared thermography could be efficiently used instead of clinical examination to detect udder health issues across lactation. Among the studied issues (asymmetry, fibrosis, abscesses, and swollen supra-mammary lymph nodes), mild and extended fibrosis were associated with lower udder skin surface temperatures, while temperature variations were observed in different stages of abscessation. Teat temperature was not a reliable indicator of chronic udder health issues, while recording season affected skin surface temperature values. Infrared thermography can be implemented within holistic udder health status assessment protocols, allowing goat farmers to improve the udder health of their animals based on evidential and informed management decisions.

## 1. Introduction

Udder-related health problems adversely affect goat health and welfare status, leading to significant production and monetary losses [1]; among them, mastitis is the most common and significant one. Mastitis is defined as the inflammation of the mammary gland, associated with intramammary infection or mechanical injury [2], and followed by relevant clinical manifestation, physiological and chemical changes in milk, and pathological alterations in the mammary gland (e.g., redness, heat, pain) [3]. Prolonged and untreated mastitis can lead to the development of chronic mastitis, associated with the occurrence of fibrotic tissue, udder asymmetry, abscesses, and secondary milk cysts within the mammary gland [4]. Therefore, systematic assessment of udder health is crucial to promptly identify udder-related issues and minimize their impact [5]. The assessment of udder health status includes clinical examination and other field or laboratory methods. Clinical examination involves both inspection and palpation of the udder tissue and is a simple and inexpensive method for the identification of udder lesions and abnormalities [6]; however, it requires restraining of the animals, it is subjective, and many farmers lack the training to carry it out effectively. Other methods utilized for the detection of intramammary infections and mastitis include bacterial culture and isolation, serological and molecular testing, estimation of the somatic cell counts, detection of inflammatory markers in milk (i.e., acute-phase protein concentration and enzymatic activity in milk), and measurement of milk electrical conductivity [7,8,9]. Yet, these methods either cannot be used at the point of care (POC) or require skilled staff and specialized equipment [10,11].

Currently, advances in information technology and computers, including artificial intelligence, deep learning models, and the Internet of Things, along with the production of low-cost devices, have accelerated the adoption of modern technologies for the monitoring of animal health and welfare as an integral part of the precision livestock farming concept [12]. Indeed, sensor-based tools can collect real-time, continuous, and objective data from animals, enabling the detection of changes related to animal health, behavior, and physiology [13], thus allowing farmers to intervene at an early stage and make informed management decisions [14]. Among these tools, ultrasonography and infrared thermography have been proposed as promising imaging technologies for the rapid assessment of udder health at the POC. Ultrasonography has been used for the accurate detection of lesions and alterations in the teat cistern, teat canal, and udder parenchyma tissues [15], as well as for the clinical study of swollen supra-mammary lymph nodes, abscesses, granulomas, and excessive fibrous tissue [16]; however, it is time-consuming and requires appropriate restraining of the animals, specialized equipment, and advanced end-user skills in handling the equipment and interpreting the results. On the contrary, infrared thermography (IRT) is a rapid, user-friendly, and proximal optical sensing technology requiring minimal animal handling and producing easily interpretable results for the end user.

In recent years, IRT has emerged in animal science as a non-invasive, precise technology for the on-site detection of heat, stress [17,18,19], estrus [20,21], nutritional and metabolic disorders [22,23], inflammatory processes, and systematic infectious diseases [24,25,26]. Infrared thermography operates based on the laws of Stefan–Boltzmann, Wien and Planck, which state that all objects with a temperature above absolute zero emit electromagnetic radiation in the infrared wavelength [27]. Changes in the tissues’ metabolic rate and blood-flow modifications observed in cases of inflammation, pain, and edema [28,29] result in variations in body temperature [30]; IRT can estimate the body temperature by quantifying the amount of thermal energy emitted by it, generating thermal images, where each pixel represents a distinct temperature value [31].

Several authors have assessed IRT’s capacity to identify mastitis, primarily in cows [32,33,34], as well as its capacity to evaluate teat stress induced by milking machines [35,36], examine the effects of overmilking [37], and compare the performance of different milking equipment [38].

In the recent work by Korelidou et al. [39], the use of IRT for the evaluation of udder health status in dairy ruminants was critically reviewed, addressing that most of the research has been mainly focused on the detection of subclinical and clinical mastitis in cattle. In this context, there have been considerable research efforts dedicated to developing algorithms and training models for the automatic detection of mastitis in cows using IRT [40,41]. Despite the advancements achieved in this field, and the promising outcomes, studies on the use of IRT in small ruminants, particularly goats, are still scarce [42,43,44].

Regular clinical examination of the udder is essential for identifying relevant health-related issues to enable timely management decisions that promote animal health and welfare and increase productivity; however, such examinations are invasive, laborious, and require specific expertise by the examiner. Given that IRT has emerged as a promising non-invasive and real-time monitoring tool, the objective of this study was to examine IRT’s capacity to be used for the rapid and in situ clinical assessment of udder health status in goats across lactation.

## 2. Materials and Methods

Two studies, a prospective and a cross-sectional one, were designed to evaluate the capacity of IRT to diagnose udder health issues across lactation and on different farms, respectively, while testing the hypothesis that udder-half skin surface temperature (USST) values were significantly different between healthy goats and goats with specific udder health issues.

### 2.1. Farm and Animals Enrolled in the Studies

For the prospective study (S1), an intensive goat farm (Farm A) in the suburbs of Athens, Greece, located 200 m above sea level was enrolled. The climate in the region is mild Mediterranean, with temperatures ranging between −4 °C (January) and 40 °C (July). A total of 106 purebred, adult Skopelos goats (between 2 and 5 years old; mean value ± standard deviation: 3.1 ± 0.70), all at the same stage of lactation (approximately 2 months postpartum), were randomly selected. Goats were fed twice daily with 1.0 to 1.2 kg of concentrates and 0.9 to 1.8 kg of alfalfa hay, adjusted according to their nutritional demands and production stage; barley straw was provided ad libitum. Goats were milked twice per day in a 2 × 24 parallel milking parlor. The average annual milk production per goat was approximately 450 kg, with an average fat and protein content equal to 4.0% and 3.6%, respectively.

For the cross-sectional study (S2), 132 randomly selected purebred Skopelos goats, aged between 2 and 5 years old (mean value ± standard deviation: 3.5 ± 0.66), from an extensive goat farm located 50 m above sea level on Skopelos island (Farm B) were involved. The climate of the area is mild Mediterranean, characterized by dry summers and rainy winters and temperatures ranging between 5 °C (January) and 34 °C (July). The animals on that farm had the same genetic background as those on Farm A in S1, since Farm A had originally and exclusively purchased all its breeding stocks from Farm B, three years before the present study. The goats were housed in basic infrastructures and grazed daily for 8 to 12 h in natural pasturelands (grasslands, woodlands, scrublands), year-round. In addition to grazing, each lactating goat received 0.5 to 1.0 kg of concentrates and 0.0 to 0.5 kg of alfalfa hay depending on the season and the productivity. Goats were hand-milked twice a day, and the average annual milk yield production per goat was approximately 350 kg, with average fat and protein contents of 4.3% and 3.9%, respectively.

### 2.2. On-Field Study Design

The goats on Farm A were prospectively monitored every 50 days, between February and August 2022 (4 recording sessions (RS); RS1 = February—20 days post-weaning, RS2 = April—70 days post-weaning, RS3 = June—mid-lactation—120 days post-weaning, RS4 = August—170 days post-weaning/end of lactation period), while S2 took place in April 2022. Thermal images were captured within 2 h after the morning milking, using a Flir E8 thermal camera (FLIR Systems Inc., Wilsonville, OR, USA) configured at 0.95 emissivity [45,46], 320 × 240 resolution, 0.05 °C thermal sensitivity, 2.6 mrad spatial resolution, and ±2 °C accuracy. Following milking, all goats remained inside the barn and each goat was individually examined; during the process, they were mildly restrained and their tail was gently raised to have a clear view of the udder. Thermal images were captured by a single trained operator at approximately 0.70 m from the udder. Before capturing thermal images, no manipulation of the udder was allowed, and it was assured that the udders were clean and dry; moreover, in all cases, the assessment site was not exposed to wind or sunlight. Following image capturing, each animal was subjected to a thorough clinical examination of the udder by the same experienced veterinarian throughout the study. The examination included visual inspection and palpation of the udder for the detection of (i) clinical mastitis (presence of pain, swelling, inflammation), (ii) udder asymmetry (one half being at least 25% longer than the other), (iii) udder fibrosis (fibrotic tissue at palpation), (iv) udder abscesses (categorized based on their position as (a) deep-seated or (b) superficial and based on their developmental stage as (a) developed, (b) fully mature, or (c) drained), (v) swollen supra-mammary lymph nodes (a 5-degree scale was used to categorize them according to their size—1: pea, 2: almond, 3: nutmeg, 4: nut, 5: mandarin or bigger; lymph nodes assigned a value >2 were considered swollen), and (vi) other udder health-related issues (udder and teat cysts, teat fibrosis, udder skin lesions, papillomatosis). Also, individual goats’ milk yield was recorded during morning milking for each RS and daily milk yield (DMY) was estimated according to the International Committee of Animal Recording (ICAR) recommendations, while the body condition score (BCS) was evaluated using a 5-point scale with 0.25 increments (1 = emaciated, 5 = obese) by a single trained and experienced veterinarian. The animals were handled according to the EU legislation for animal welfare. The experimental procedure was approved under number 33/08.06.21, issued by the Ethics Committee of the Agricultural University of Athens.

### 2.3. Thermal Images and Statistical Analyses

**Figure 1:**
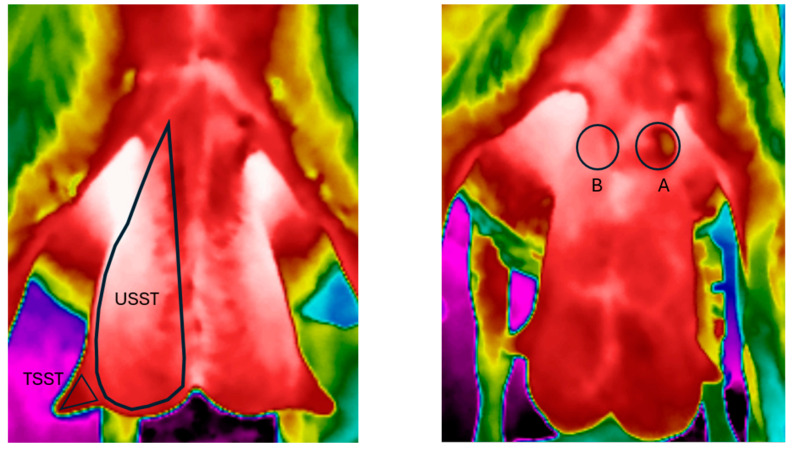
Visualization of the udder skin surface temperature of each udder half (USST), the teat skin surface temperature (TSST), and the skin surface temperature of an abscess (A) and its symmetrical healthy area (B) (source: Laboratory of Anatomy and Physiology of Farm Animals, Agricultural University of Athens).

Thermal images illustrating the udder half and teat skin surface temperatures (USST and TSST, respectively) were analyzed using the FLIR Thermal Studio Software (FLIR Systems Inc., Wilsonville, OR, USA). The polyline tool was used to outline the margins of the udder halves and teats to record the minimum (min), mean, and maximum (max) temperatures of the skin surface in these areas. During outlining, attention was given to exclude any area in contact with the inner part of the thighs that might have influenced the results. The ellipse tool was used to delineate the limits of the abscesses in the affected halves, and the estimated temperatures were compared with the respective symmetrical area of the healthy udder half. Each udder half was treated as a separate unit for the statistical analyses (Figure 1). Only the cases where the udder half was either healthy or exhibited a single health issue, such as fibrosis, asymmetry, swollen mammary lymph nodes, or a combination of udder fibrosis and asymmetry, were considered (group 0 (G0) = healthy, group 1 (G1) = presence of only fibrosis, group 2 (G2) = presence of both fibrosis and asymmetry, group 3 (G3) = presence of only asymmetry, group 4 (G4) = presence of swollen supra-mammary lymph nodes). Udder halves with multiple health issues, those with fewer than two cases of specific udder health issues, as well as halves with teats or abscesses positioned on the front side of the udder were not considered for further analyses. For the study of abscesses, cases from both Farm A and Farm B were jointly considered and analyzed.

Statistical analyses were performed using SPPS v.26 (IBM Corp., Armonk, NY, USA). The point prevalence values of the recorded udder health issues were estimated by counting, while descriptive statistics were used to calculate the max, mean, and min USST and TSST values per udder health issue as well as per recording session (RS). For normally distributed temperature values, differences between sampling occasions and different health issues were analyzed using one-way ANOVA followed by Tukey’s post hoc analysis for pairwise comparisons, while in cases where temperature values were not normally distributed, the Kruskal–Wallis test was used instead. Finally, a mixed linear regression model was used to assess the effects of the occurrence of the studied udder health issues on the maximum, mean, and minimum USST and TSST, after adjusting for the recording session, as presented below (Model 1):

T_gj_ = μ + R_j_ + U_gj_ + γ_j_ + e_gj_ (1)

where: T_gj_ = maximum, mean, and minimum USST and TSST values (°C) for the gth recording session of the jth goat; μ = intercept; R_j_ = fixed effect of the recording session (4 levels; 1st–4th recording session); U_gj_ = fixed effect of the udder half health status (5 levels; 0 = healthy, 1 = presence of fibrosis, 2 = presence of both fibrosis and asymmetry, 3 = presence of asymmetry, 4 = presence of swollen supra-mammary lymph nodes); γ_j_ = repeated variation of the jth goats’ udder halves; and e_gj_ = residual error. Statistical significance was set at the 0.05 level, while a statistical significance level of 0.001 was used when necessary.

## 3. Results

### 3.1. Descriptive Statistics

Table 1 presents the point prevalence values (number of goats exhibiting a particular udder health-related issue at a specific point of time/total number of goats at that specific point of time) of the studied udder health-related issues in all the animals enrolled in S1 and S2 from both Farm A and Farm B. In Farm A, point prevalence values varied across the four RSs. In particular, the point prevalence of either fibrosis, asymmetry, or both increased from 11.0 to 20.8%, 14.0 to 19.8%, and 4.0 to 11.3%, respectively, by RS3 and then slightly decreased to 20.4, 15.5, and 6.8%, respectively, in RS4. The point prevalence values of abscesses were constantly low during the study, ranging from 0.9 to 2.0%, while for swollen mammary lymph nodes, it fluctuated throughout the RSs, displaying decreased values in RS2 (1.9%) and RS4 (1.0%) and a slight increase in RS3 (7.5%). No clinical cases of acute mastitis were found during the RSs throughout the study, while the prevalence of other udder health issues (udder and teat cysts, teat fibrosis, udder skin lesions, papillomatosis) was critically low to be further considered for the analyses. Daily milk yield in RS1 was 2.0 ± 0.76 kg and decreased to 1.1 ± 0.47 kg in RS4, while the mean BCS was within the desired range and fluctuated between 2.9 and 3.2 during the study.

In Farm B, 60.6% of goats displayed no udder health issues. The point prevalence values of fibrosis, asymmetry, both fibrosis and asymmetry, swollen supra-mammary lymph nodes, and abscesses were 22.7, 16.7, 6.8, 11.4, and 6.8%, respectively. In this farm, the average DMY was 1.8 ± 0.54 kg and the average BCS was 2.9 ± 0.22.

### 3.2. Thermal Image Analyses—Temperature Values

#### 3.2.1. Udder and Teat Skin Surface Temperature Values on Farm A

**Figure 2:**
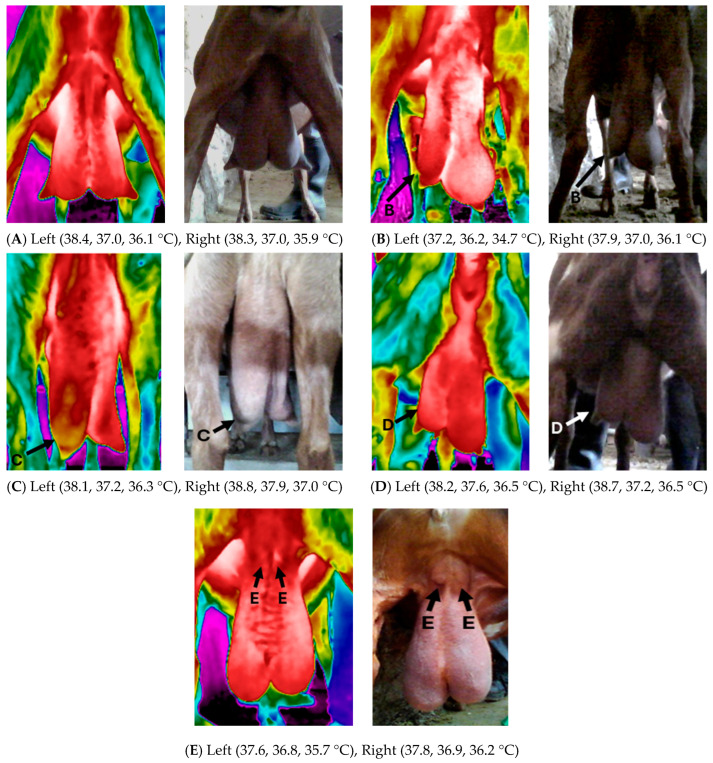
Thermal images of different udder health conditions: (**A**) healthy udder halves, (**B**) fibrotic and asymmetric left udder half, (**C**) fibrotic left udder half, (**D**) asymmetric left udder half, (**E**) swollen supra-mammary lymph nodes in both udder halves. Maximum, mean, and minimum udder skin surface temperature of each udder half ((**Left**) and (**Right**)) are presented below the images (source: Laboratory of Anatomy and Physiology of Farm Animals, Agricultural University of Athens).

Out of the 200, 208, 212, and 206 total udder halves included in this study in RS1, RS2, RS3, and RS4, respectively, only 196 udder halves and 165 teats were considered for further analyses in RS1, 205 udder halves and 183 teats in RS2, 210 udder halves and 193 teats in RS3, and 204 udder halves and 200 teats in RS4, following the predefined exclusion criteria. Table 2 and Table 3 present the max, mean, and min USST and TSST of each udder half according to their health status (Figure 2) per RS on Farm A. Udder skin surface temperature values varied significantly between RS in G0, displaying an increase of 1.2, 2.2, and 3.3 °C, in the max, mean, and min USST, respectively, in RS4. The lowest variations in the max and mean USST were observed in G2, with increases of 0.7 °C and 1.5 °C, respectively, in RS3, followed by a decrease of 0.3 °C in RS4. In contrast, G1 displayed the highest variations, with an increase of 1.7, 3.0, and 4.8 °C for the max, mean, and min USST, respectively. G4 exhibited the highest increase in the max and mean USST by RS2 (1.1 and 1.3 °C, respectively), although no clear temperature patterns were observed in G3 and G4. Additionally, between G0 and G1 the highest temperature differences were observed in RS1 (0.5, 0.7, and 1.3 °C for the max, mean, and min USST, respectively), which steadily decreased by RS4. Contrarily, between G0 and G2 the differences in the max and mean USST were the lowest in RS1 (0.4 and 0.6 °C, respectively) and increased in RS4 to 1.2 and 1.6 °C, respectively. The max, mean, and min TSST of G0 significantly increased by 4.3, 4.9, and 5.7 °C, respectively, while the smallest variations were noticed again in G2, where the temperature increased by 3.3, 3.7, and 5.0 °C, respectively, across recordings. No significant differences were observed in the mean and max TSST among the groups, except for the min TSST of G2, which was significantly lower than G1 in RS4 (*p* < 0.05). G2 displayed the lowest USST values among all the groups throughout the entire lactation period except for the mean and the max USST in RS1 (mean and max USST were 0.1 °C higher than G1). Specifically, it was noticed that when comparing USST values per RS, the max, mean, and min USST in G2 were lower by 0.4–1.2 °C, 0.6–1.6 °C, and 0.8–2.1 °C, respectively, compared to G0; 0.1–1.1 °C, 0.2–1.8 °C, and 0.5–2.2 °C, respectively, compared to G3; 0.0–0.9 °C, 0.3–1.5 °C, and 0.9–1.9 °C, respectively, compared to G4; and 0.2–1.2 °C, 0.3–1.7 °C, and 0.4–1.8 °C, respectively, compared to G1. Indeed, G2 displayed significantly lower mean and min USST in RS3 compared to G0 and G4 (*p* < 0.05) and the lowest mean USST among all the groups in RS4 (*p* < 0.05).

#### 3.2.2. Udder and Teat Skin Surface Temperature Values on Farm B

Out of the 264 udder halves initially considered in this study, only 241 udder halves and 206 teats were further analyzed. Table 4 presents the variations in the min, mean, and max USST and TSST of udder halves based on their health status on Farm B in S2. G2 displayed the lowest max, mean, and min USST among all groups; in particular, these values were lower by 0.4, 0.4, and 0.9 °C, respectively, compared to G0; by 0.2, 0.4, and 0.6 °C, respectively, compared to G1; by 0.3, 0.3, and 1.0 °C, respectively, compared to G3; and by 0.4, 0.4, and 1.3 °C, respectively, compared to G4. However, no statistically significant differences were observed in the min, mean, and max TSST and USST among the groups.

#### 3.2.3. Skin Surface Temperature at the Abscess Site

**Figure 3:**
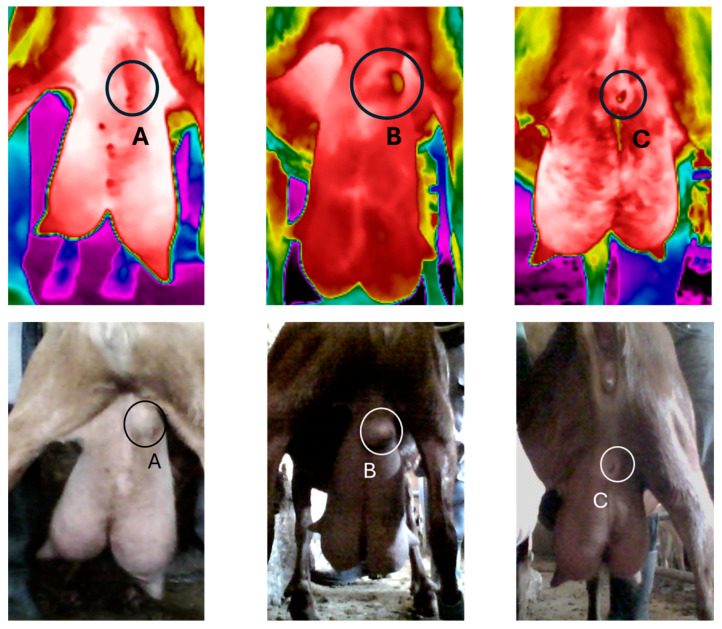
Types of abscesses based on their developmental stage: superficial developed (A), superficial fully mature (B), and drained (C) (source: Laboratory of Anatomy and Physiology of Farm Animals, Agricultural University of Athens).

Thirteen out of a total of 17 cases of abscesses identified in 15 goats (superficial developed, superficial fully mature, and drained) were further analyzed (Figure 3). Table 5 illustrates the skin surface temperature (SST) differences at the abscess site and the symmetrical site of the healthy udder half observed among the different types of abscesses, as identified during the clinical examination of goats on both Farms A and B. Superficial developed abscesses displayed temperature changes in the affected area, with the max, mean, and min SST values being lower by 0.8, 1.1, and 1.2 °C, respectively, compared to the symmetrical site of the healthy halves. When abscesses fully matured, the lowest temperature value was observed at the tip of the abscess (34.8 °C compared to 36.4 °C at the symmetrical site of the healthy udder half). The mean temperature was also lower by 0.8 °C compared to the healthy areas, while no difference was observed in the max temperature. Drained abscesses demonstrated a cold visible scar surrounded by higher temperature values (32.0 and 36.3 °C, respectively); mean SST did not differ, but max SST was 0.7 °C higher than the healthy tissue.

### 3.3. Effect of Udder Health Status on Teat and Udder Skin Surface Temperature

Table 6 and Table 7 summarize the effects of the RS and the udder health status on the max, mean, and min TSST and USST, respectively. The RS was associated with a consistent and statistically significant decrease in both TSST and USST when compared to the reference category (RS4) in all cases (*p* < 0.001). Additionally, udder health status seemed to have a statistically significant effect on USST; in particular, fibrosis was associated with a decrease of 0.2, 0.3, and 0.3 °C on the max, mean, and min USST, respectively, (*p* < 0.05), while the presence of both fibrosis and asymmetry resulted in a decrease of 0.6, 0.9, and 1.4 °C on the max, mean, and min USST, respectively (*p* < 0.001). On the contrary, udder asymmetry and swollen lymph nodes were not found to be significantly associated with USST. Finally, TSST was not affected by udder health status in any case.

## 4. Discussion

Herein, we present for the first time the potential of IRT to be exploited for the in situ clinical assessment of udder health status and for the study of temperature variations in udder halves with clinical findings of chronic mastitis in goats. Towards this objective, a prospective study was carried out to evaluate temperature fluctuations in the USST of goats with different udder health issues across lactation, while a cross-sectional study was used to validate the observed temperature patterns in goats using a second farm. It was found that the presence of fibrosis, especially when it was accompanied by asymmetry, resulted in a significant decrease in the max, mean, and min USST, while specific temperature patterns were observed for different types of abscesses according to their stage of development. These findings showcase the potential use of IRT in the development of diagnostic protocols for the remote detection of mild and severe cases of udder fibrosis, which might otherwise be underdiagnosed, and potentially for the allocation of abscesses in the udder parenchyma. Contrarily, TSST was not evidenced to offer reliable information regarding the studied udder health-related issues, with temperature readings being mostly dependent on the recording season.

### 4.1. Udder Skin Surface Temperature and Udder Health Status

Several studies in various animal species have acknowledged the positive correlation between USST and udder health status, utilizing for this reason either somatic cell count (SCC) or California mastitis test (CMT) scoring in milk samples [27,45,47]. For example, Gayathri et al. [48] found that udder halves diagnosed with subclinical and clinical mastitis had higher USST compared to the healthy ones, increased by 0.7–1.9 °C and 1.4–2.9 °C, respectively, in crossbred goats (Alpine–Beetal and Saanen–Beetal) and irrespective of the season. In cows, similar studies concluded that subclinical mastitis leads to increased USST [27,45] by 2.4 °C [32], 1.0–1.5 °C [49], 0.9–3.0 °C [47], and 1.5 °C [50], suggesting the use of IRT as a reliable tool for the detection of mastitis [51]. The factors associated with increased USST are not fully elucidated, although the defense mechanisms of the udder, stimulated by the pathogen invasion and the resulting inflammatory response, offer a reasonable explanation evidenced by the vasodilation and the increased blood flow [52]. Inflammation is externally manifested by elevated temperature, redness, and swelling of the udder [53].

Compared to the acute inflammatory responses, prolonged and persistent inflammation leads to infiltration of mononuclear cells (macrophages, lymphocytes, and plasma cells), tissue destruction, and replacement of the damaged tissue with connective tissue, leading to fibrosis [54]. Fibrosis is characterized by the accumulation of extracellular matrix components (such as collagen and fibronectin), abnormal proliferation of fibroblasts, and replacement of the mammary gland parenchyma with fibrotic tissue [55]. In severe cases, chronic mastitis leads to the shrinkage or complete destruction of alveoli with the extended replacement of healthy tissue with fibrotic tissue, resulting in structural, functional, and morphological changes (e.g., asymmetry of the udder halves) [56]. The aforementioned changes doubled with the impaired vascular functionality, and the reduced blood flow and oxygen supply [57] of the mammary gland provide a reasonable explanation for the lowering of USST values observed in groups G1 and G2.

The highest difference in USST values between G0 and G1 was observed in RS1 and steadily decreased by RS4. It is known that the stage of lactation is associated with modifications in the blood flow within the mammary gland, with the latter normally decreasing after reaching the peak of lactation [56], which was also the case in our study. Consequently, the normally reduced blood flow in RS4 while preparing for the transition to the dry period might have resulted in comparable USST values in groups G0 and G1. On the contrary, when G0 was compared to G2, the lowest difference in USST values was observed at the first stage of lactation (RS1), with a gradually increasing trend by the end of lactation (RS4). Advanced lesions of the untreated, severe, chronic mastitis cases in G2, characterized by extensive fibrosis and followed by impaired blood circulation, could justify the more evident temperature differences over time.

Udder asymmetry without underlying fibrosis was not associated with changes in the USST. Udder asymmetry may result from various factors, including genetic ones [58], unilateral suckling [16], and subclinical intramammary bacterial infections (e.g., by coagulase-negative staphylococci) [58] or other chronic infections (e.g., by Mycoplasma spp. and lentiviruses) [59]. In our study, the occurrence of asymmetry associated with non-pathogenic factors could explain the absence of significant USST temperature decreases in asymmetric udder halves.

Swollen supra-mammary lymph nodes were not found to be associated with USST changes. During an active immunological response in the udder, lymphocytes in the ipsilateral supra-mammary lymph nodes are activated, proliferate, and migrate into the mammary gland to combat invading pathogens [60]. This reaction results in lymphatic circulation modifications and morphological changes in the supra-mammary lymph nodes of the affected animals, which present as swollen [55]. In our study, we evaluated the USST variation across the entire udder halves, rather than the supra-mammary lymph node region; the lack of significant USST differences between groups could be indicative of mild subclinical inflammation in the udder (i.e., subclinical mastitis). Additionally, the relatively low number of udder halves with swollen supra-mammary lymph nodes did not facilitate the drawing of safe conclusions.

### 4.2. Teat Skin Surface Temperature

None of the studied udder health-related issues was significantly associated with changes in TSST; however, a tendency was observed in the case of the co-existence of udder fibrosis and asymmetry. Although TSST in cows has been evaluated as a potential indicator of clinical and subclinical mastitis with varying and, in many cases, controversial results, relevant studies on goats remain scarce. According to Gayathri et al. [48], TSST was higher in goats with subclinical and clinical mastitis irrespective of the season, which is consistent with the findings by Satheesan at al. [45] and Gayathri et al. [61] in Sahiwal cows (udder quarters with subclinical and clinical mastitis displayed higher TSST by 1.7–3.2 °C and 0.6–2.3 °C, respectively) [61]. Notably, TSST in the Furstenberg’s rosette sphincter area was 9.6 °C and 4.6 °C higher in cows with mastitis compared to healthy cows at ambient temperatures below and above 0 °C, respectively [62]. In Murrah buffaloes, TSST at different levels (apex, barrel, base) showed a positive correlation with SCC [63]. On the contrary, in other studies, TSST either was not correlated with SCC [44] or demonstrated low diagnostic performance in detecting mastitis [64] and poor discriminating capacity regarding mastitis-causing pathogens [65].

In cases of persistent infections, the progression of the inflammatory processes and the reduced blood flow due to decreased udder functionality may result in low TSST [65]. In goats, chronic mastitis has been associated with udder atrophy, thickened teat walls, and narrowed teat canals [66]. Therefore, the tendency of G2 to exhibit lower TSST could be attributed to the chronicity of lesions, the presence of fibrous tissue, and impaired blood circulation at the teats. However, the lack of statistical significance could be linked to the confounding effect of the season of recording and mainly the ambient temperatures the teats were exposed at.

In general, temperature distribution within the udder demonstrates remarkable differences, with the highest SST values being observed in the upper parts of the udder [64]. In contrast, the teat tip appears to be consistently the coldest part of the udder in both goats [35,44] and cows [37]. This indicates a higher dependency of TSST on the ambient temperatures or reduced blood circulation at the teat tip [44,64]. Indeed, ambient temperature has been shown to significantly affect the SST of the teat sphincter in healthy cows (*p* < 0.01) [62]. Our study aligns with the findings of Saathesan et al. and Kittur et al. [45,63], where TSST consistently remained lower than the other USST values across all RSs, regardless of the udder health status.

### 4.3. Abscesses

This study demonstrated that superficial developed abscess sites displayed lower SST values compared to the symmetric sites of the healthy udder halves. Superficial fully mature abscesses exhibited lower mean and min SST but similar values of the max temperature, while drained abscesses had higher temperatures at their periphery, lower temperatures at the draining site, and no difference in the mean temperature compared to healthy tissue of the respective symmetrical site. To our knowledge, this is the first time infrared thermography has been used to detect the margins and types of abscesses based on the SST variation and distribution thereof. Despite the low number of abscesses studied, the findings seem to be consistent with the pathogenetic mechanisms leading to udder abscess formation. Indeed, abscess formation is a defense mechanism elicited by the host to restrict and eradicate the pathogens invading the udder causing chronic intramammary infections and mastitis [67,68]; during the initial stages of the abscess development, the invading pathogens trigger an inflammatory response and the recruitment of immune cells to the infection site [69]. Fibroblastic proliferation, cellular organization and repair processes at the affected tissue margins, and encapsulation of abscesses by granulation and fibrotic tissue development follow during later developmental stages [55]. The abscess wall thickness varies based on the location, depth, and chronicity of infection; chronic and the deep-seated abscesses have thicker walls compared to superficial ones [70]. The formation of a thick abscess pseudocapsule and the replacement of sufficiently vascularized healthy tissue by necrotic tissue and pus are likely to account for the low temperature values observed in superficial developed and superficial fully mature abscesses in our study. During their maturation, abscesses increase in size, eventually rupture and release pus [71], and heal by scarring [54]. Increased internal pressure along with the thinning of the overlying skin before rupturing [72] may have caused the slight reduction in the max temperature difference between healthy tissue and superficial fully mature abscesses observed in our study. Once the abscess ruptures, the wound-healing process initiates. The lower temperature observed at the abscess tip can be attributed to the presence of fibrotic scar, while the increased temperature at the periphery of the tip was most likely due to the angiogenesis observed during the healing process and the resulting increased blood flow [73].

### 4.4. Environmental Conditions and Study Setup

In our study, the RSs significantly influenced max, mean, and min USST and TSST values, which is a rather expected finding associated with the changes in the environmental conditions inside the barn. On Farm A, by RS4, the mean ambient temperature inside the barn had risen by 18 °C, contributing to the observed increase in the USST. Other environmental conditions such as ambient humidity, solar radiation, and airflow are also known to influence the body and udder temperature in farm animals and are likely to have contributed to the changes observed in USST and TSST in our study, although minimal exposure to environmental conditions was considered during the study design and implementation stages [74]. Similar season-dependent variations in USST and TSST have been observed in other ruminants as well [75,76]. For example, in cows, elevated ambient temperatures during the hot season have been associated with increased body and udder temperatures compared to the cold season (by approximately 1.0 °C) [50], when the low ambient temperatures trigger vasoconstriction, reducing blood flow in the superficial blood vessels to minimize heat loss [77].

Other animal-related parameters, such as the circadian rhythm, stress, feeding pattern, and physical activity, may also influence the diagnostic performance of thermal imaging [78]. To account for the effects of the aforementioned factors, thermal images were consistently captured at the same time, post-milking, ensuring appropriate handling of animals to avoid stress. Additionally, images were collected from 0.70 m, which is within the range described in similar previous studies (0.30–2.50 m) [40,41,78,79,80]. Dirt, urine, fecal and bedding material on the udder can reduce USST; however, udder cleaning by rubbing or using water can directly affect USST [51]. Thus, no manipulation of the udder was performed before capturing the thermal images, and only thermal images of goats with clean udders were finally considered to be further analyzed. Finally, the average USST and TSST ranges of healthy goats in this study (35.3–37.5 °C and 32.0–36.9 °C, respectively) differed from those reported by Gayathri et al. [48] (33.9–38.3 °C and 33.6–38.2 °C, respectively), verifying the potential effects of breed-related factors, climatic conditions, time of the day, and thermal camera settings for thermal image acquisition.

In our study, thermal images were analyzed after the manual alignment of the udder and teat borders using the polygon tool to estimate the max, mean, and min udder and teat SST of the selected areas. Currently, there is no standard segmentation protocol for the analysis of the thermal images of goat udders, and even in cows the most common approach includes selecting a circular and centrally located area over the teat [49]. Among mean, min, and max USST, studies have shown that max USST exhibits the highest correlation with rectal temperature [81] and better diagnostic performance compared to the average temperature [79]. Metzner et al. [30] demonstrated that the highest difference between healthy udders and udders with mastitis was observed when the max temperature was utilized using the polygon and rectangle tools, while Stumpf et al. [81] recommended using a horizontal triangle on the lateral side of the udder to segment the udder within thermal image and estimate the max USST. Herein, the highest temperature differences were observed when using the min temperature, which is reasonable considering the chronic nature of the observed udder health issues; however, utilizing minimum values can be a challenging task, as this value can be extensively influenced by the presence of dirt, manure, and urine and the excessive hair coverage in some cases. Therefore, a careful selection of the analyzed area is necessary to avoid artificially induced USST outliers due to confounding factors.

### 4.5. Future Prospectives and Suggestions

Currently, udder fibrosis is typically detected by udder palpation, which requires relevant training and experience by the examiner, which many farmers lack. The lower USST values observed in the udder halves with severe fibrosis indicate that IRT could be a practical tool for the farmer to differentiate between asymmetric udder halves accompanied by fibrosis and those without any underlying pathological condition. This can significantly enhance efficient udder health management at the herd level, where underdiagnosed or untreated mastitis leading to fibrosis may be associated with significant monetary and production losses and compromised animal welfare. Moreover, modern, portable, and low-cost thermal cameras can be connected to smartphones, and with the proper settings and operator training, they can be used as an auxiliary tool for farmers to evaluate the udder health status of their animals in an objective, rapid, user-friendly, and real-time manner, allowing for informed management decisions. In addition, the distinct patterns among temperature distributions observed between different types of abscesses in thermal images based on their developmental stage suggest the potential application of IRT for monitoring the abscess maturation process, while the site with the lowest temperature in a mature abscess may be indicative of the most suitable site for the skin incision, minimizing the risk of excessive bleeding.

This study focused only on udder halves exhibiting either asymmetry, fibrosis, or a combination of the two, as well as swollen supra-mammary lymph nodes, excluding cases where multiple issues co-existed within the same udder half. The idea was to assess whether IRT could be used as a tool alternative to clinical examination to diagnose clinically manifested udder health-related issues. The inclusion of more cases from various farms and the inclusion of other health-related issues that were not observed in our study, such as udder skin lesions, teat fibrosis, and clinical mastitis, are expected to facilitate a better understanding of the temperature variation based on the udder health status, therefore emerging as crucial future objectives towards a more comprehensive approach considering the monitoring of the udder health status via IRT. Additionally, although the association between subclinical mastitis, somatic cell counts, and the USST distribution was beyond the scope of the present study, it always forms a significant research challenge in the field of the POC diagnostics of mastitis.

Finally, in our study, the recording season seemed to influence temperature readings, while the analysis of the thermal images was performed manually using software and selecting the whole area of the udder and teats. Future studies should consider automated procedures for either segmenting the udder or choosing targeted areas on it to correlate the USST values with various udder health-related issues. Moreover, integrating environmental conditions into prediction models could enhance the accuracy of assessments. The lack of standardized protocols for capturing and analyzing thermal images along with the various animal- and environment-related factors that affect the accuracy of measurements are among the most important drawbacks that need to be addressed; the development of advanced algorithms and the integration of deep learning techniques to adjust for the aforementioned factors could significantly improve the applicability of IRT by enhancing the accuracy and reliability of IRT-based diagnostics in the future [82].

In any case, our study can be used as a reference for the USST pattern in goats with different chronic udder health issues during lactation, and as a stepping stone to further assess the potential utilization of IRT for the reliable and proximal non-invasive clinical diagnosis of specific udder health issues.

## 5. Conclusions

This is the first study to investigate the skin surface temperatures of udders and teats demonstrating different clinically manifested chronic udder issues across lactation in goats. Mild and severe cases of fibrosis in goats significantly decreased udder skin surface temperature, rendering infrared thermography a candidate tool to be utilized for the diagnosis of fibrotic udders, after relevant validation efforts. Also, the distinct temperature patterns according to the type of udder abscesses imply the potential use of IRT for the non-invasive, contactless, and proximal allocation and characterization of abscesses according to their developmental stage. However, the high dependency of the udder skin surface temperature readings on the recording season and factors associated with animals’ physiology and operational variation render necessary the implementation of further studies to determine the most crucial relevant adjustments in future udder health assessment protocols when considering the application of IRT.

## Figures and Tables

**Table 1:** Point prevalence values of the studied udder health-related issues, mean daily milk yield, and body condition score values (± standard deviation) per recording session, including all the goats in Farms A and B.

	Farm A				Farm B *
	RS1 (N = 100)	RS2 (N = 104)	RS3 (N = 106)	RS4 (N = 103)	(N = 132)
Healthy (%)	73.0	67.3	64.2	70.9	60.6
Abscesses (%)	2.0	1.9	0.9	1.0	6.8
Fibrosis (%)	11.0	19.2	20.8	20.4	22.7
Asymmetry (%)	14.0	17.3	19.8	15.5	16.7
Swollen mammary lymph nodes (%)	8.0	1.9	7.5	1.0	11.4
Both fibrosis and asymmetry (%)	4.0	4.8	11.3	6.8	6.8
Daily milk yield (kg)	2.0 ± 0.76	1.5 ± 0.62	1.5 ± 0.67	1.1 ± 0.47	1.8 ± 0.54
Body condition score (1–5)	2.9 ± 0.19	3.2 ± 0.33	3.2 ± 0.40	3.1 ± 0.35	2.9 ± 0.22

* Assessments on Farm B were conducted at the same season and stage of lactation as those of the 2nd recording session on Farm A (RS2). RS1 = February—20 days post-weaning, RS2 = April—70 days post-weaning, RS3 = June—mid-lactation—120 days post-weaning, RS4 = August—170 days post-weaning/end of lactation period.

**Table 2:** Average maximum, mean, and minimum teat skin surface temperature values (mean ± standard deviation) based on the udder half health status across lactation on Farm A.

	RS1	RS2	RS3	RS4
**Number of teats considered for the analysis (n)**	165	183	193	200
Healthy	141	151	155	166
Fibrosis	5	13	14	18
Fibrosis and asymmetry	3	5	9	5
Asymmetry	8	12	11	11
Swollen supra-mammary lymph nodes *	8	2	4	-
**Maximum teat skin surface temperature (°C)**				
Healthy	33.0 ± 1.15 ^a, 1^	34.3 ± 1.04 ^a, 2^	36.0 ± 0.64 ^a, 3^	37.3 ± 0.67 ^a, 4^
Fibrosis	33.7 ± 0.68 ^a, 1^	33.9 ± 0.81 ^a, 1^	35.9 ± 0.90 ^a, 2^	37.5 ± 0.57 ^a, 3^
Fibrosis and asymmetry	33.1 ± 0.87 ^a, 1^	33.4 ± 1.12 ^a, 1^	35.8 ± 0.83 ^a, 2^	36.4 ± 1.69 ^a, 2^
Asymmetry	32.9 ± 1.11 ^a, 1^	33.7 ± 1.24 ^a, 1^	35.8 ± 0.51 ^a, 2^	37.5 ± 0.41 ^a, 3^
Swollen supra-mammary lymph nodes	33.3 ± 1.61 ^a, 1^	34.0 ± 0.92 ^a, 1^	35.8 ± 0.19 ^a, 1^	NA
**Mean teat skin surface temperature (°C)**				
Healthy	32.0 ± 1.13 ^a, 1^	33.1 ± 1.02 ^a, 2^	35.3 ± 0.64 ^a, 3^	36.9 ± 0.75 ^a, 4^
Fibrosis	32.3 ± 0.46 ^a, 1^	32.9 ± 0.79 ^a, 1^	35.3 ± 0.82 ^a, 2^	37.1 ± 0.62 ^a, 3^
Fibrosis and asymmetry	32.3 ± 1.10 ^a, 1^	32.4 ± 1.12 ^a, 1^	35.1 ± 0.67 ^a, 2^	36.0 ± 1.71 ^a, 2^
Asymmetry	31.6 ± 1.10 ^a, 1^	32.7 ± 0.93 ^a, 2^	35.2 ± 0.40 ^a, 3^	37.0 ± 0.50 ^a, 4^
Swollen supra-mammary lymph nodes	32.0 ± 1.68 ^a, 1^	33.8 ± 0.92 ^a, 1, 2^	35.1 ± 0.31 ^a, 2^	NA
**Minimum teat skin surface temperature (°C)**				
Healthy	30.6 ± 1.20 ^a, 1^	31.8 ± 1.26 ^a, 2^	34.6 ± 0.78 ^a, 3^	36.3 ± 0.91 ^a, b, 4^
Fibrosis	30.0 ± 1.39 ^a, 1^	31.9 ± 0.85 ^a, 2^	34.5 ± 0.82 ^a, 3^	36.7 ± 0.64 ^a, 4^
Fibrosis and asymmetry	30.2 ± 1.95 ^a, 1^	31.4 ± 1.26 ^a, 1^	34.4 ± 0.69 ^a, 2^	35.2 ± 1.56 ^b, 2^
Asymmetry	30.1 ± 1.48 ^a, 1^	31.7 ± 0.88 ^a, 2^	34.5 ± 0.70 ^a, 3^	36.4 ± 0.59 ^a, b, 4^
Swollen supra-mammary lymph nodes	30.6 ± 1.84 ^a, 1^	33.2 ± 1.27 ^a, 1, 2^	34.3 ± 0.54 ^a, 2^	NA

NA: not applicable. RS1 = February—20 days post-weaning, RS2 = April—70 days post-weaning, RS3 = June—mid-lactation—120 days post-weaning, RS4 = August—170 days post-weaning/end of lactation period. Healthy = G0, fibrosis = G1, fibrosis and asymmetry = G2, asymmetry = G3, swollen supra-mammary lymph nodes = G4. The ambient temperature and humidity values inside the barn were 12 °C and 70% (RS1), 18 °C and 56% (RS2), 26 °C and 34% (RS3), and 30 °C and 38% (RS4). For each of the studied teat skin surface temperature values, different letter superscripts (^a–b^) within the same column (and separately for minimum, mean, and maximum temperature) indicate significant differences between health status groups. Different numerical superscripts (^1–4^) within the same row indicate significant differences between recording sessions within the same health status group (*p* < 0.05). * In RS4, only 1 goat had both supra-mammary lymph nodes swollen; however, the respective udder halves and teats were both excluded from further analyses due to the co-existence of other udder health issues.

**Table 3:** Average maximum, mean, and minimum udder skin surface temperature values (mean ± standard deviation) based on the udder half health status across lactation in Farm A.

	RS1	RS2	RS3	RS4
**Number of udder halves considered for the analysis (n)**	194	205	210	204
Healthy	166	171	166	170
Fibrosis	5	14	15	18
Fibrosis and asymmetry	4	5	10	5
Asymmetry	10	13	11	11
Swollen supra-mammary lymph nodes	9	2	8	-
**Maximum udder skin surface temperature (°C)**				
Healthy	37.2 ± 0.76 ^a, 1^	37.6 ± 0.74 ^a, 2^	38.1 ± 0.60 ^a, 3^	38.4 ± 0.59 ^a, 4^
Fibrosis	36.7 ± 0.95 ^a, 1^	37.2 ± 0.55 ^a, 1^	37.8 ± 0.63 ^a, 2^	38.4 ± 0.64 ^a, 2^
Fibrosis and asymmetry	36.8 ± 0.90 ^a, 1^	37.0 ± 1.00 ^a, 1^	37.5 ± 0.46 ^a, 1^	37.2 ± 1.53 ^a, 1^
Asymmetry	36.9 ± 0.97 ^a, 1^	37.3 ± 1.04 ^a, 1^	37.8 ± 0.47 ^a, 1, 2^	38.3 ± 0.49 ^a, 2^
Swollen supra-mammary lymph nodes	36.8 ± 0.66 ^a, 1^	37.9 ± 0.00 ^a, 2^	38.0 ± 0.30 ^a, 2^	NA
**Mean udder skin surface temperature (°C)**				
Healthy	35.3 ± 0.77 ^a,1^	35.7 ± 0.89 ^a, 2^	37.0 ± 0.58 ^a, 3^	37.5 ± 0.65 ^a, 4^
Fibrosis	34.6 ± 0.96 ^a, 1^	35.1 ± 0.72 ^a, 1^	36.7 ± 0.77 ^a, b, 2^	37.6 ± 0.75 ^a, 2^
Fibrosis and asymmetry	34.7 ± 1.69 ^a, 1^	34.8 ± 1.50 ^a, 1^	36.2 ± 0.42 ^b, 1^	35.9 ± 1.78 ^b, 1^
Asymmetry	35.2 ± 0.91 ^a, 1^	35.0 ± 0.84 ^a, 1^	36.8 ± 0.65 ^a, b, 2^	37.7 ± 0.51 ^a, 3^
Swollen supra-mammary lymph nodes	35.0 ± 0.65 ^a, 1^	36.3 ± 0.21 ^a, 2^	37.0 ± 0.40 ^a, 2^	NA
**Minimum udder skin surface temperature (°C)**				
Healthy	33.1 ± 1.07 ^a, 1^	34.0 ± 0.87 ^a, 2^	35.9 ± 0.52 ^a, 3^	36.4 ± 1.24 ^a, 4^
Fibrosis	31.8 ± 1.03 ^a, 1^	33.5 ± 0.77 ^a, 2^	35.5 ± 0.71 ^a, b, 3^	36.6 ± 0.91 ^a, 4^
Fibrosis and asymmetry	31.0 ± 1.92 ^a, 1^	32.3 ± 2.36 ^a, 1, 2^	35.1 ± 0.51 ^b, 2^	34.8 ± 1.80 ^a, 2^
Asymmetry	33.2 ± 1.01 ^a, 1^	33.2 ± 1.02 ^a, 1^	35.6 ± 0.77 ^a, b, 2^	36.7 ± 0.64 ^a, 3^
Swollen supra-mammary lymph nodes *	32.5 ± 1.56 ^a, 1^	34.2 ± 0.28 ^a, 1, 2^	36.0 ± 0.44 ^a, 2^	NA

RS: recording session; NA: not applicable. RS1 = February—20 days post-weaning, RS2 = April—70 days post-weaning, RS3 = June—mid-lactation—120 days post-weaning, RS4 = August—170 days post-weaning/end of lactation period. Healthy = G0, fibrosis = G1, fibrosis and asymmetry = G2, asymmetry = G3, swollen supra-mammary lymph nodes = G4. The ambient temperature and humidity values inside the barn were 12 °C and 70% (RS1), 18 °C and 56% (RS2), 26 °C and 34% (RS3), and 30 °C and 38% (RS4). For each of the studied udder skin surface temperature values, different letter superscripts (^a–b^) within the same column (and separately for minimum, mean, and maximum temperature) indicate significant differences between health status groups. Different numerical superscripts (^1–4^) within the same row indicate significant differences between recording sessions within the same health status group (*p* < 0.05). * In RS4, only 1 goat had both supra-mammary lymph nodes swollen; however, the respective udder halves and teats were both excluded from further analyses due to the co-existence of other udder health issues.

**Table 4:** Average maximum, mean, and minimum teat and udder skin surface temperature values, based on the udder half health status on Farm B.

	Healthy	Fibrosis	Fibrosis and Asymmetry	Asymmetry	Swollen Supra-Mammary Lymph Nodes
Number of teats considered for the analysis (n)	167	16	7	9	7
Number of udder halves considered for the analysis (n)	193	21	8	10	9
Maximum teat skin surface temperature (°C)	34.8 ± 1.04	34.6 ± 1.28	34.9 ± 1.30	34.9 ± 0.94	34.9 ± 0.68
Mean teat skin surface temperature (°C)	34.0 ± 1.04	34.0 ± 1.40	34.0 ± 1.59	34.0 ± 1.06	34.2 ± 0.71
Minimum teat skin surface temperature (°C)	32.8 ± 1.22	32.8 ± 1.57	32.5 ± 1.72	32.5 ± 1.48	33.2 ± 0.84
Maximum udder skin surface temperature (°C)	38.1 ± 0.76	37.9 ± 1.09	37.7 ± 0.65	38.0 ± 0.53	38.1 ± 0.63
Mean udder skin surface temperature (°C)	36.4 ± 0.86	36.4 ± 1.10	36.0 ± 1.08	36.3 ± 0.65	36.4 ± 0.42
Minimum udder skin surface temperature (°C)	34.7 ± 0.98	34.4 ± 0.85	33.8 ± 0.93	34.8 ± 0.70	35.1 ± 0.51

Healthy = G0, fibrosis = G1, fibrosis and asymmetry = G2, asymmetry = G3, swollen supra-mammary lymph nodes = G4. Ambient temperature and humidity level during this study were 17 °C and 63%. No statistically significant differences in udder and teat skin surface temperature values were observed between the studied udder health status-based groups.

**Table 5:** Average maximum, mean, and minimum skin surface temperature values in the regions of the abscess development and the symmetrical regions of the healthy udder halves in goats of Farms A and B.

Abscess Type Based on the Developmental Stage	Maximum (°C)	Mean (°C)	Minimum (°C)
**Superficial developed** (*n* = 9)	36.4 ± 0.57	35.1 ± 1.09	34.0 ± 1.40
Paired healthy area in the symmetrical udder half	37.2 ± 0.83	36.2 ± 1.12	35.2 ± 1.25
**Superficial fully mature** (*n* = 2)	37.2 ± 0.85	36.1 ± 0.80	34.8 ± 0.30
Paired healthy area in the symmetrical udder half	37.2 ± 0.95	36.9 ± 0.85	36.4 ± 0.90
**Drained** (*n* = 2)	36.3 ± 1.30	34.8 ± 1.20	32.0 ± 1.95
Paired healthy area in the symmetrical udder half	35.6 ± 1.15	34.8 ± 1.35	33.6 ± 1.20

**Table 6:** Effects of the recording session and the udder health status on the maximum, mean, and minimum teat skin surface temperature values (°C).

Parameter	Category Level	B	SE	*p*-Value	95% CI	
					Lower	Upper
Maximum teat skin surface temperature (°C)						
Intercept		37.3	0.07	<0.001	37.2	37.5
Sampling	RS1	−4.3	0.09	<0.001	−4.5	−4.1
	RS2	−3.2	0.09	<0.001	−3.3	−3.0
	RS3	−1.3	0.08	<0.001	−1.5	−1.2
	RS4			Ref		
Udder health status	Fibrosis	0.0	0.13	0.790	−0.3	0.2
	Fibrosis and asymmetry	−0.4	0.20	0.054	−0.8	0.0
	Asymmetry	−0.1	0.14	0.417	−0.4	0.2
	Swollen supra-mammary lymph nodes	0.2	0.23	0.506	−0.3	0.6
	Healthy			Ref		
Mean teat skin surface temperature (°C)						
Intercept		36.9	0.07	<0.001	36.8	37.0
Sampling	RS1	−4.9	0.09	<0.001	−5.1	−4.7
	RS2	−3.9	0.09	<0.001	−4.1	−3.7
	RS3	−1.6	0.08	<0.001	−1.7	−1.4
	RS4			Ref		
Udder health status	Fibrosis	0.0	0.13	0.853	−0.3	0.2
	Fibrosis and asymmetry	−0.4	0.20	0.074	−0.7	0.0
	Asymmetry	−0.1	0.14	0.404	−0.4	0.2
	Swollen supra-mammary lymph nodes	0.2	0.24	0.398	−0.3	0.7
	Healthy			Ref		
Minimum teat skin surface temperature (°C)						
Intercept		36.3	0.08	<0.001	36.2	36.5
Sampling	RS1	−5.8	0.11	<0.001	−6.0	−5.5
	RS2	−4.5	0.10	<0.001	−4.7	−4.3
	RS3	−1.8	0.10	<0.001	−2.0	1.6
	RS4			Ref		
Udder health status	Fibrosis	0.0	0.15	0.908	−0.3	0.3
	Fibrosis and asymmetry	−0.4	0.23	0.064	−0.9	0.0
	Asymmetry	−0.1	0.17	0.587	−0.4	0.2
	Swollen supra-mammary lymph nodes	0.2	0.28	0.411	−0.3	0.8
	Healthy			Ref		

CI: confidence interval; B: coefficient; SE: standard error; Ref: reference category. RS1 = February—20 days post-weaning, RS2 = April—70 days post-weaning, RS3 = June—mid-lactation—120 days post-weaning, RS4 = August—170 days post-weaning/end of lactation period. Healthy = G0, fibrosis = G1, fibrosis and asymmetry = G2, asymmetry = G3, swollen supra-mammary lymph nodes = G4.

**Table 7:** Effects of the recording session and the udder health status on the maximum, mean, and minimum udder skin surface temperature values.

Parameter	Category Level	B	SE	*p*-Value	95% CI	
					Lower	Upper
Maximum udder skin surface temperature (°C)						
Intercept		38.4	0.05	<0.001	38.3	38.5
Sampling	RS1	−1.3	0.07	<0.001	−1.4	−1.1
	RS2	−0.8	0.07	<0.001	−1.0	−0.7
	RS3	−0.3	0.06	<0.001	−0.5	−0.2
	RS4			Ref		
Udder health status	Fibrosis	−0.2	0.10	0.015	−0.4	0.0
	Fibrosis and asymmetry	−0.6	0.15	<0.001	−0.9	−0.4
	Asymmetry	−0.2	0.11	0.070	−0.4	0.1
	Swollen supra-mammary lymph nodes	−0.1	0.15	0.554	−0.4	0.2
	Healthy			Ref		
Mean udder skin surface temperature (°C)						
Intercept		37.6	0.05	<0.001	37.4	37.7
Sampling	RS1	−2.3	0.07	<0.001	−2.4	−2.2
	RS2	−1.9	0.07	<0.001	−2.1	−1.8
	RS3	−0.6	0.06	<0.001	−0.7	−0.5
	RS4			Ref		
Udder health status	Fibrosis	−0.3	0.10	0.001	−0.5	−0.1
	Fibrosis and asymmetry	−0.9	0.16	<0.001	−1.3	−0.6
	Asymmetry	−0.2	0.11	0.135	−0.4	0.1
	Swollen supra-mammary lymph nodes	0.0	0.16	0.866	−0.3	0.4
	Healthy			Ref		
Minimum udder skin surface temperature (°C)						
Intercept		36.5	0.07	<0.001	36.4	36.6
Sampling	RS1	−3.5	0.10	<0.001	−3.7	−3.3
	RS2	−2.5	0.10	<0.001	−2.7	−2.4
	RS3	−0.5	0.09	<0.001	−0.7	−0.3
	RS4			Ref		
Udder health status	Fibrosis	−0.3	0.14	0.014	−0.6	−0.1
	Fibrosis and asymmetry	−1.4	0.21	<0.001	−1.8	−1.0
	Asymmetry	−0.2	0.15	0.169	−0.5	0.1
	Swollen supra-mammary lymph nodes	−0.1	0.23	0.534	−0.6	0.3
	Healthy			Ref		

CI: confidence interval; B: coefficient; SE: standard error; Ref: reference category. RS1 = February—20 days post-weaning, RS2 = April—70 days post-weaning, RS3 = June—mid-lactation—120 days post-weaning, RS4 = August—170 days post-weaning/end of lactation period. Healthy = G0, fibrosis = G1, fibrosis and asymmetry = G2, asymmetry = G3, swollen supra-mammary lymph nodes = G4.

## Data Availability

Data available upon request.

## Abbreviations

The following abbreviations are used in this manuscript:

B	Coefficient
BCS	Body condition score
CI	Confidence interval
CMT	California mastitis test
SCC	Somatic cell count
DMY	Daily milk yield
G0	Healthy group
G1	Only fibrotic udder halves group
G2	Both fibrotic and asymmetric udder halves group
G3	Only asymmetric udder halves group
G4	Only swollen supra-mammary lymph nodes group
IRT	Infrared thermography
Max	Maximum
Min	Minimum
POC	Point of care
Ref	Reference
RS	Recording session
RS1	Recording session 1
RS2	Recording session 2
RS3	Recording session 3
RS4	Recording session 4
S1	Study 1
S2	Study 2
SE	Standard error
SST	Skin surface temperature
TSST	Teat skin surface temperature
USST	Udder skin surface temperature
